# On Commensalism of *Candida*

**DOI:** 10.3390/jof6010016

**Published:** 2020-01-17

**Authors:** Jesus A. Romo, Carol A. Kumamoto

**Affiliations:** Department of Molecular Biology and Microbiology, Tufts University, Boston, MA 02111, USA; Jesus.Romo@Tufts.edu

**Keywords:** *Candida*, gastrointestinal, colonization, mycobiome, commensalism, adhesion

## Abstract

*Candida* species are both opportunistic fungal pathogens and common members of the human mycobiome. Over the years, the main focus of the fungal field has been on understanding the pathogenic potential and disease manifestation of these organisms. Therefore, understanding of their commensal lifestyle, interactions with host epithelial barriers, and initial transition into pathogenesis is less developed. In this review, we will describe the current knowledge on the commensal lifestyle of these fungi, how they are able to adhere to and colonize host epithelial surfaces, compete with other members of the microbiota, and interact with the host immune response, as well as their transition into opportunistic pathogens by invading the gastrointestinal epithelium.

## 1. Introduction

*Candida* species are opportunistic fungal pathogens and common members of the human mycobiome [1,2,3]. *Candida* species, primarily *Candida albicans*, are early colonizers acquired at or near birth primarily by physical contact [4,5]. These organisms are able to colonize the skin [6], as well as the gastrointestinal [7,8], and reproductive tracts of humans [9,10]. *C. albicans* is the most studied member of the genus with most of the research focused on its ability to cause disease [7,11,12,13,14,15]. Indeed, *C. albicans* is responsible for an unacceptably high number of symptomatic infections yearly that range from superficial (i.e., skin and mucous membranes) to invasive (i.e., internal organs) [7,16,17]. Most of these infections occur in immunocompromised individuals and originate from the gastrointestinal tract [18,19,20,21,22,23]. Therefore, it is crucial to narrow the gap in knowledge involving colonization, commensal lifestyle, and transition into a pathogenic state by *Candida* species. Interestingly, *Candida* species also appear to carry out functions that benefit the host. These include playing a role in the development of mucosal immune responses [24,25] and protection from *Clostridioides difficile* infection in a murine model [26]. *Candida* species have clearly developed an intimate relationship with the host, which benefits the fungus and the host under certain circumstances. When this relationship is disturbed by antibiotic treatment or immune suppression however, the results can be catastrophic to the host. Below, we describe the current knowledge of *Candida* colonization and commensal lifestyle, with a brief discussion of invasion of epithelial barriers, primarily in the gastrointestinal tract.

## 2. *Candida* Gastrointestinal Colonization

As mentioned above, *Candida* species are acquired during or near birth [4,5] and become a significant part of the host mycobiome [1,2,3,27,28,29,30]. In order to cement their place in the host, they must be able to adhere to host epithelial cells and mucosal surfaces, resist and interact with host immune responses, and compete or cooperate with other members of the host microbiota. Additionally, *C. albicans* possesses the ability to undergo filamentation, a morphogenetic change from yeast to hyphae in response to an array of environmental signals, many of which are found in the host gastrointestinal tract [31]. Hyphae are composed of elongated cells that do not separate after division and are the pathogenic form due to their ability to invade tissues. Hyphae are also required for proper biofilm formation of *C. albicans*, which allows it to endure environmental insults (e.g., antifungal treatment) [31,32,33,34,35]. Therefore, in order to successfully colonize the gastrointestinal tract, *C. albicans* must also successfully orchestrate morphogenetic transitions without harming the host and being eliminated by the subsequent immune response.

### 2.1. Adhesion

To date, there are no detailed studies of *Candida* species in the intestinal environment that strictly focus on commensalism. Moreover, knowledge of the role of adhesins during *Candida* colonization of the intestinal tract is minimal [36,37]. In this sub-section we will discuss the current knowledge of adhesins and their role in other niches that *Candida* species occupy and their potential role during intestinal colonization. The most studied adhesins utilized by *C. albicans* belong to the agglutinin-like sequence (ALS) gene family [38,39,40], which encode a group of GPI-anchored proteins with adhesive properties. These proteins have previously been shown to be present on the surface of the fungal cell wall [41,42], a structure composed mainly of chitin, glucans (β-1,3 and β-1,6), and mannans [43,44,45,46,47,48]. The ALS gene family consists of eight members (*ALS1*-*ALS7* and *ALS9*), with *ALS1* and *ALS3* being the most studied. Distinct members of the ALS family are expressed during the yeast and hyphal morphologies of *C. albicans* [49,50,51]. *ALS3* and the non-ALS adhesin Hyphal Wall Protein 1 (*HWP1*) are expressed mainly by hyphae [39,49,50,52,53,54]. Indeed, the distinct array of adhesins might be required for adaptation to distinct niches occupied by *Candida* species (i.e., oral, intestinal, and vaginal mucosal surfaces). Early studies have shown that *C. albicans* exhibits a greater ability to adhere to buccal epithelial cells (BECs) [55,56], vaginal epithelial cells (VECs) [57,58,59], uroepithelial cells [60], epithelium derived cell monolayers [61,62,63], and intestinal epithelial cells in vitro compared to other *Candida* species [64,65,66]. Although these studies did not directly address the role of specific adhesins, more recent studies have begun to elucidate specific roles [39,49,52,64,67,68,69,70,71,72]. Zakikhany et al., showed that the hyphal specific adhesin, *ALS3*, is highly upregulated during epithelial infection in vitro and that deletion of *ALS3* decreases this adhesion [73]. Similarly, deletion of *ALS2*, decreases adhesion [74,75]. Importantly, deletion of *ALS5*, *ALS6*, or *ALS7* resulted in an increase in adhesion highlighting the complex roles of adhesins [76]. Studies with *HWP1* have also demonstrated that this hyphal specific adhesin is highly expressed during colonization and infection of the oral epithelium and its deletion attenuates virulence in a murine model of oropharyngeal candidiasis [51,73,77]. The current knowledge of the role of adhesins in tissue culture systems and animal models is summarized in Table 1. These observations combined with the fact that *C. albicans* is able to very successfully colonize the gastrointestinal tract of humans [8,78,79,80], suggest that indeed distinct adhesins are required to successfully interact with the different environmental niches encountered by *C. albicans* and that multiple adhesins might be required for intestinal colonization. 

*C. albicans* morphogenetic states might play distinct roles during colonization of the gastrointestinal tract. *C. albicans* cells in the yeast state display decreased adhesion to enterocytes compared to oral epithelial cells in vitro [87]. Moreover, in these studies, most *C. albicans* cells underwent morphological transitions into hyphae, which enhanced their adhesive properties, as well as their virulence due to hyphal invasion into the host epithelial surfaces [50,87]. *C. albicans* invasion of epithelial surfaces occurs via two well characterized mechanisms, active penetration and endocytosis, both briefly described below. These in vitro studies suggest that *C. albicans* initial adhesion triggers filamentation and expression of virulence factors which lead to invasion of an epithelium. *C. albicans* must establish a relationship with the host in which none or minimal damage is caused in order to establish itself as a long-term colonizer [88]. When *C. albicans* is acquired early in life there could be some initial damage due to filamentation, which could be overcome after the host immune response is able to mature and control pathogenic fungal populations.

*C. albicans* hyphal specific transcripts have been detected in several studies of murine models of gastrointestinal colonization [36,89]. The role of hyphae in intestinal commensalism however is still enigmatic since some studies have reported that yeast is the predominant morphology colonizing the gastrointestinal tract (~90%) [89,90]. More recently, Witchley and co-workers were able to show that yeast and hyphae morphologies co-occur throughout the gastrointestinal tract in a murine model. More importantly, they were able to identify a regulatory program involved in balancing commensal and pathogenic lifestyles of *C. albicans* [37]. The authors demonstrated that a master regulator of *C. albicans* filamentation, Ume6, reduced intestinal colonization fitness by activating the expression of the secreted aspartic proteinase 6 (Sap6) as well as the adhesin Hyr1, which triggered a pro-inflammatory response and subsequent clearance of the fungus by the host immune response. These results highlight the role of the environmental pressure coming from the host immune response and the ability of *C. albicans* populations to adapt to this stress. 

To date, the majority of the studies involving adhesion and morphological states of *C. albicans* have been performed using in vitro models and primarily host niches other than the lower gastrointestinal tract. Clearly, the role of morphogenetic states along with specific adhesins expressed has not been fully addressed in the context of intestinal colonization and more studies are required in order to fully understand the role of these distinct morphologies, in addition to adhesins in interactions with host epithelial barriers.

In the last few decades, isolation of non-*Candida albicans Candida* (NCAC) species has significantly increased [5,13,91,92,93,94]. Less is known about NCAC as colonizers of the gastrointestinal tract. *C. glabrata* is another opportunistic pathogenic yeast and successful colonizer of the human gastrointestinal tract [1,2,3,28]. Although it is classified under the genus *Candida*, it is not a member of the CUG clade and is more closely related to *Saccharomyces cerevisiae* than to *C. albicans* [95]. *C. glabrata* is the second most commonly isolated *Candida* species from human samples in clinical laboratories behind *C. albicans* [91,96,97,98,99,100,101]. *C. glabrata* is unable to form true hyphae, but it is capable of adhering to host epithelial surfaces [102]. The adhesin repertoire utilized by *C. glabrata* consists of Epithelial Adhesin (Epa) and Epa-like proteins. Of this adhesin family, Epa1p has been shown to mediate adhesion to host epithelial cells (human laryngeal carcinoma, Hep2) [102] and macrophages [103]. Moreover, Epa6 and Epa7 have been shown to moderate adhesion to epithelial and endothelial cells [104,105]. In the case of the intestinal tract, a previous study identified adhesion properties of specific adhesins of *C. glabrata* expressed in *S. cerevisiae* to human epithelial colorectal adenocarcinoma cells (Caco2). The authors found that the adhesive properties of different adhesins ranged from significant to very weak adherence with Epa1, 6, and 7 displaying significant to moderate adhesion and Epa19-21 displaying weak adhesion [106]. *C. glabrata* colonization of the gastrointestinal tract is not well understood. Previous findings suggest that *C. glabrata* may require *C. albicans* for colonization and invasion of the oral cavity. Tati and co-workers demonstrated that *C. albicans* Als1 and Als3 were required for in vitro *C. glabrata* adhesion to *C. albicans* hyphae and to establish oropharyngeal candidiasis (OPC) in a murine model [107]. Moreover, *C. glabrata* cell wall protein genes *EPA8*, *EPA19*, *AWP2*, *AWP7* and *CAGL0F00181*, were required for adhesion to *C. albicans*. These studies demonstrate that *C. glabrata* might be dependent on *C. albicans* for adhesion and invasion in the oral environment. Are these examples of fungal interactions that are important in the intestinal tract? In a recent interesting study, Gonia and co-workers demonstrated that another NCAC, *C. parapsilosis*, which is primarily a fungal pathogen of neonates, was able to protect premature intestinal epithelial cells (pIECs) from *C. albicans* invasion and damage [108]. More importantly, this protection was correlated with the adhesiveness of *C. parapsilosis* to *C. albicans* and to pIECs. These studies demonstrate that NCACs might play distinct roles during *C. albicans* colonization, invasion, and pathogenesis and further studies are required to begin to understand the complex ecosystem in which these fungi exist and interact with each other.

### 2.2. Specialized Morphotype for Survival in the Gastrointestinal Tract

More recently, *C. albicans* Gastrointestinally indUced Transition (GUT) cells were identified by allowing wild type *C. albicans* to pass through the mammalian gastrointestinal tract [109]. These cell types underwent a phenotypic switch due to the expression of *WOR1*, a master switch transcription factor of white-opaque phenotypic switching in *C. albicans* [110,111]. White-opaque switching has been well characterized [112]. White and opaque cells have distinct gene expression of a large variety of processes [113,114]. Moreover, opaque cells have been identified as the mating competent phase [115,116]. Interestingly, GUT cells proved to be hypercompetitive and more fit in gastrointestinal colonization models as well as less virulent in a bloodstream murine model relative to white cells [109]. These observations indicate that yeast and hyphae are not the only morphogenetic states involved in colonization and commensalism of the host gastrointestinal tract. Moreover, GUT cells also express an array of transcripts optimized for lower gastrointestinal tract colonization. Among these, genes involved in the use of fatty acids and *N*-acetyl-glucosamine were upregulated, while genes involved in adhesion, glucose utilization, and iron uptake were downregulated. These patterns indicate that GUT cells are highly adapted to the nutrients available in the host lower gastrointestinal tract. Interestingly, GUT cells share similar metabolic characteristics with opaque cells, which are attenuated for commensalism [113]. These novel morphotypes require more investigation in order to truly understand their role in gastrointestinal colonization. What role do GUT cells play in *C. albicans* colonization and commensalism and do other *Candida* species have specialized cell types that promote gastrointestinal colonization?

### 2.3. Effects of C. albicans Colonization during Bacterial Infection

As mentioned earlier, most of the research on *Candida* species has focused on their pathogenic potential and there is a gap in knowledge about potential benefits provided to the host. One interesting example comes from murine studies performed by our group [26]. In these studies, antibiotic treated mice were either not colonized or pre-colonized with *C. albicans.* The animals were then challenged with the bacterial pathogen *Clostridioides difficile.* Surprisingly, the mice that were pre-colonized with *C. albicans* displayed a higher survival rate of a lethal *C. difficile* infection compared to the non-colonized mice. Moreover, one mechanism by which this protection was conferred was by the upregulation of *IL-17A* during *C. albicans* colonization. 

Several groups have identified and characterized the interactions between *C. albicans* and *Pseudomonas aeruginosa* in distinct niches [117,118,119]. The interactions between these two organisms appear to be primarily antagonistic [117,120,121,122]. Previously, Lopez-Medina and co-workers used a neutropenic murine model to investigate the effect of *C. albicans* gastrointestinal colonization on *P. aeruginosa* pathogenesis [123]. Surprisingly, *C. albicans* specifically inhibited *P. aeruginosa* virulence without affecting the bacterium’s colonization capabilities. The authors were able to determine that *C. albicans* inhibited *P. aeruginosa* virulence by suppressing gene expression of the siderophores pyochelin and pyoverdine. Moreover, the inhibition of these two siderophores by *C. albicans* increased mouse survival and decreased *P. aeruginosa* dissemination highlighting the importance of mycobiome in host health and disease. Thus, *C. albicans* colonization reduced disease caused by different bacterial pathogens. 

Conversely, *C. tropicalis* has been shown to play a role in the exacerbation of Crohn’s disease (CD) [124]. Hoarau and co-workers analyzed the micro- and mycobiota of patients with CD and their healthy relatives and found the bacteria *Serratia marcescens* and *Escherichia coli* elevated in CD patients. Interestingly, *C. tropicalis* was also elevated in CD patients and its abundance positively correlated with *S. marcescens* and *E. coli*, suggesting potential interactions between these three organisms. In subsequent experiments, the authors described an in vitro biofilm composed of all three organisms, which showed enhanced biomass, a distinct morphology of *C. tropicalis* in contrast to *C. tropicalis* only biofilms, and physical interactions between the organisms. 

These examples indicate that related fungi may occupy distinct ecological roles in the host gastrointestinal tract. There is a need for further examination of the role of fungal commensals during host health and disease. 

### 2.4. Host Immune Response

The host is constantly surveying mucosal surfaces for pathogens and commensals such as *C. albicans*. Previous studies have demonstrated that the host is able to distinguish between yeast and filamentous *C. albicans* and therefore is able to identify commensalism or pathogenic infections [125]. Additionally, the host is able to sense increased fungal burdens, which could indicate an active infection, and respond [126]. For a review on specific receptors utilized by the host to sense fungi, see references [8,127]. Among these, Dectin-1 has been shown to be crucial for controlling fungal populations and both mice and humans either lacking or having a distinct polymorphism are highly prone to ulcerative colitis presumably triggered by fungal overgrowth [128]. Previous studies have suggested that *C. albicans* is able to select populations for colonization based on the immune status of the host [36,89]. In these studies, the authors demonstrated that *C. albicans* expression of the transcription factor Efh1p is upregulated in cells colonizing the murine gastrointestinal tract. Interestingly, expression of *EFH1* is associated with reduced colonization efficiency of the intestinal tract in animal models. In these models, cells lacking *EFH1* are able to successfully colonize the intestinal tract at higher levels than wild type, which displays intermediate colonization capabilities, while overexpression of *EFH1*, resulted in poor colonization of the intestinal tract. These studies suggest that *C. albicans* might be able to regulate population size in the host by modifying its gene expression, which could prevent strong immune responses by the host and subsequent antifungal effects. Another interesting adaptation of *C. albicans* in order to colonize the intestinal tract, is the expression of the transcription factor *EFG1*. This major regulator of *C. albicans* morphogenesis regulates the yeast to hyphal transition by responding to a variety of stimuli [84,129,130,131,132,133]. Previous studies demonstrated that expression of *EFG1* by *C. albicans* in the intestinal tract differed depending on the immune status of the host [134]. In contrast to the studies performed with *EFH1*, *C. albicans efg1*^−^ null mutant proved to be hypersusceptible to the host immune response and therefore a poor colonizer. Both of these studies demonstrate that *C. albicans* gene expression is intimately connected to host immune status and this ability to accurately and effectively sense the host allows *C. albicans* to be a successful colonizer.

### 2.5. Host Microbiota

In the host, *Candida* also has to compete for space and nutrients with other microorganisms in the ecosystem. Previous studies showed that anaerobes suppress *C. albicans* growth in the gastrointestinal tract of Syrian hamsters and that the removal of those anaerobes by treatment with penicillin allowed *C. albicans* to adhere, colonize, and disseminate from the gastrointestinal tract [135,136]. More recently, there is emerging evidence that members of the host microbiota affect *C. albicans* colonization of the gastrointestinal tract. One such study demonstrated that *Lactobacillus* species are antagonistic to *C. albicans* by producing indole-3-aldehyde (IAld), which stimulated IL-22 production resulting in antifungal activity [137]. Cruz and co-workers identified a EntV, a peptide secreted by *Enterococcus faecalis*, which was shown to inhibit *C. albicans* filamentation and virulence in a nematode infection model [138,139]. In these studies, the authors demonstrated that co-infection of a nematode with *E. faecalis* and *C. albicans* resulted in less pathology and less mortality than infection with either organism alone. The author hypothesizes that this phenomenon promotes a mutually beneficial association with the host, leading to a commensal lifestyle [140]. More recently, the same group demonstrated that EntV alone reduces invasion, inflammation, and fungal burden in a murine model of oropharyngeal candidiasis [139]. Lastly, Fan and co-workers were interested in factors that allow mice to exhibit *C. albicans* colonization resistance [141]. The authors identified a key role for commensal anaerobic bacteria, mainly clostridial Firmicutes and Bacteroidetes, in which the bacteria are able to activate the hypoxia-inducible factor-1α (HIF-1α). This activation led to the induction of the antimicrobial peptide LL-37 (CRAMP), which in murine models, resulted in significant reduction of *C. albicans* intestinal colonization and a 50% decrease in mortality from invasive disease. These studies are outstanding examples of the complex interactions that *C. albicans* has to negotiate in order to colonize the host intestinal tract and highlight how dynamic and adaptable *C. albicans* must be in order to colonize distinct host niches. 

Although *Candida* can establish an intimate and primarily benign relationship with the host, disturbances caused by antibiotics [21,142], damage of host epithelial barriers (e.g., due to bacterial toxins, trauma, or surgery) [143,144,145], or immune suppression [16] can lead to *Candida* overgrowth, invasion, and a transition to a pathogenic state.

## 3. Invasion

*C. albicans* invasion of epithelial surfaces leads to pathogenesis and is dependent on the yeast to hypha transition [146]. Interestingly, non-filamentous fungi such as *C. glabrata* have been shown to cross epithelial barriers in vitro [147]. Conversely, an efg1- null mutant displays a reduced ability to cross an epithelial Caco2 monolayer compared to the wild type and complemented strain [148]. These two observations highlight potentially distinct mechanisms of transepithelial migration and invasion. *C. albicans* invasion has been well characterized and two main mechanisms have emerged. These include epithelial-driven endocytosis [50,73,87,149] and *C. albicans* active penetration of epithelial surfaces by hyphae [12,87,150]. In vitro models have demonstrated that active penetration is the primary method of invading enterocytes during early stages of interaction [87]. Interestingly, recent studies have suggested that endocytosis occurs at a later time after *C. albicans* adheres to cells and this could be a potential mechanism for translocation through the gut barrier [151]. Both of these mechanisms have been extensively reviewed by Basmaciyan and co-workers [152]. Interestingly, Als3p has been reported to play a role in both endocytosis, active penetration, and overall invasion [146]. Results from these studies demonstrate that *C. albicans* as a commensal can interact with host epithelial barriers in distinct ways which allow it to colonize, invade, or both.

## 4. Concluding Remarks

During colonization, *C. albicans* interacts with its bacterial co-colonizers, the host epithelium and effectors of the host immune system. The outcome of these many interactions determines whether *C. albicans* will colonize as a benign commensal or become an invasive pathogen. Future work will reveal more details of these interactions, shedding light on the impact of *C. albicans* on the gastrointestinal tract ecosystem.

## Figures and Tables

**Table 1:** *C. albicans* adhesin profile in distinct niches.

Adhesin	Cell Culture		Human Samples and Animal Models (Gene Expression)		References
	Gene expression studies ^a^	Adhesion assays ^b^	Human samples ^a^	Animal studies ^a^	
*ALS1*	Reconstituted human vaginal epithelium (RHVE) from A431 cell line; Human Umbilical Vein Endothelial cells (HUVEC)	FaDu (pharynx carcinoma); Human Umbilical Vein Endothelial cells (HUVEC); Oral reconstituted human epithelium (TR146 cell line)		Vaginal candidiasis; Intestinal colonization (cecum);	[36,74,81,82,83]
*ALS2*	Reconstituted human vaginal epithelium (RHVE) from A431 cell line	Human Umbilical Vein Endothelial cells (HUVEC); Oral reconstituted human epithelium (TR146 cell line)		Vaginal candidiasis	[75,81,83]
*ALS3*	Pig liver infection (ex vivo); Oral reconstituted human epithelium (TR146 cell line); Reconstituted human vaginal epithelium (RHVE) from A431 cell line; Blood	FaDu (pharynx carcinoma); Human umbilical vein endothelial cells (HUVEC); Buccal epithelial cells (BEC); Buccal reconstituted human epithelium (RHE) model of oral candidiasis	Blood	Intraperitoneal infection model (liver); Vaginal candidiasis; Tail vein (blood infection model); Colonization model (stomach, cecum, and large Intestine)	[37,73,74,81,82,84,85,86]
*ALS4*		Human Umbilical Vein Endothelial cells (HUVEC);			[75]
*ALS5*		FaDu (pharynx carcinoma)			[82,83]
*ALS8*	Blood				[86]
*ALS9*	Reconstituted human vaginal epithelium (RHVE) from A431 cell line; Human umbilical vein endothelial cells (HUVEC)			Intraperitoneal infection model (liver); Vaginal candidiasis	[75,81]
*HWP1*	Pig liver infection (ex vivo); Oral reconstituted human epithelium (TR146 cell line); Blood, Enterocytes		Blood	Intraperitoneal infection model (liver); Blood, Colonization model (stomach, cecum, and large Intestine)	[37,73,81,84,86]
*HYR1*	Oral reconstituted human epithelium (TR146 cell line); Blood			Colonization model (stomach, cecum, and large Intestine)	[37,73,86]

Table lists ^a^ adhesins highly expressed in distinct model systems (e.g., cell culture vs. in vivo) as well as ^b^ adhesins required for binding to specific cell types in adhesion assays.
